# Conjunctival Melanoma: Genetic and Epigenetic Insights of a Distinct Type of Melanoma

**DOI:** 10.3390/ijms20215447

**Published:** 2019-10-31

**Authors:** Ernesto Rossi, Giovanni Schinzari, Brigida Anna Maiorano, Monica Maria Pagliara, Alessandro Di Stefani, Emilio Bria, Ketty Peris, Maria Antonietta Blasi, Giampaolo Tortora

**Affiliations:** 1 Medical Oncology, Fondazione Policlinico Universitario Agostino Gemelli IRCCS, 00168 Rome, Italy; giovanni.schinzari@unicatt.it (G.S.); brigim@hotmail.it (B.A.M.); emilio.bria@unicatt.it (E.B.); giampaolo.tortora@policlinicogemelli.it (G.T.); 2 Medical Oncology, Università Cattolica del Sacro Cuore, 00168 Rome, Italy; 3 Ophtalmology, Fondazione Policlinico Universitario Agostino Gemelli IRCCS, 00168 Rome, Italymariaantonietta.blasi@unicatt.it (M.A.B.); 4 Dermatology, Fondazione Policlinico Universitario Agostino Gemelli IRCCS, 00168 Rome, Italy; alessandro.distefani@gmail.com (A.D.S.); ketty.peris@unicatt.it (K.P.)

**Keywords:** conjunctival, melanoma, *BRAF*, *NRAS*, *NF1*, EZH2, miRNA, genetic

## Abstract

Conjunctival melanoma (CjM) is a rare, primary cancer of the ocular region. Genetic and epigenetic characteristics of conjunctival melanoma have not been completely elucidated yet. Conjunctival melanoma presents similarities with cutaneous melanoma, with substantial differences in the biological behavior. We reviewed the genetic and epigenetic insights of CjM involved in invasion and metastatic spread. CjM is commonly characterized by mutations of v-raf murine sarcoma viral oncogene homolog B1 (*BRAF*), neurofibromin 1 (*NF1*) and telomerase reverse transcriptase (*TERT*), high expression of mammalian target of rapamycin (mTOR) and heat shock protein 90 (HSP90), frequent phosphatase and tensin homolog (PTEN) loss and upregulation of specific miRNAs. These features should identify CjM as a distinct subset of melanoma with its own profile, which is more similar to cutaneous melanoma than mucosal melanoma and remarkably different from uveal melanoma.

## 1. Introduction

**Figure 1:**
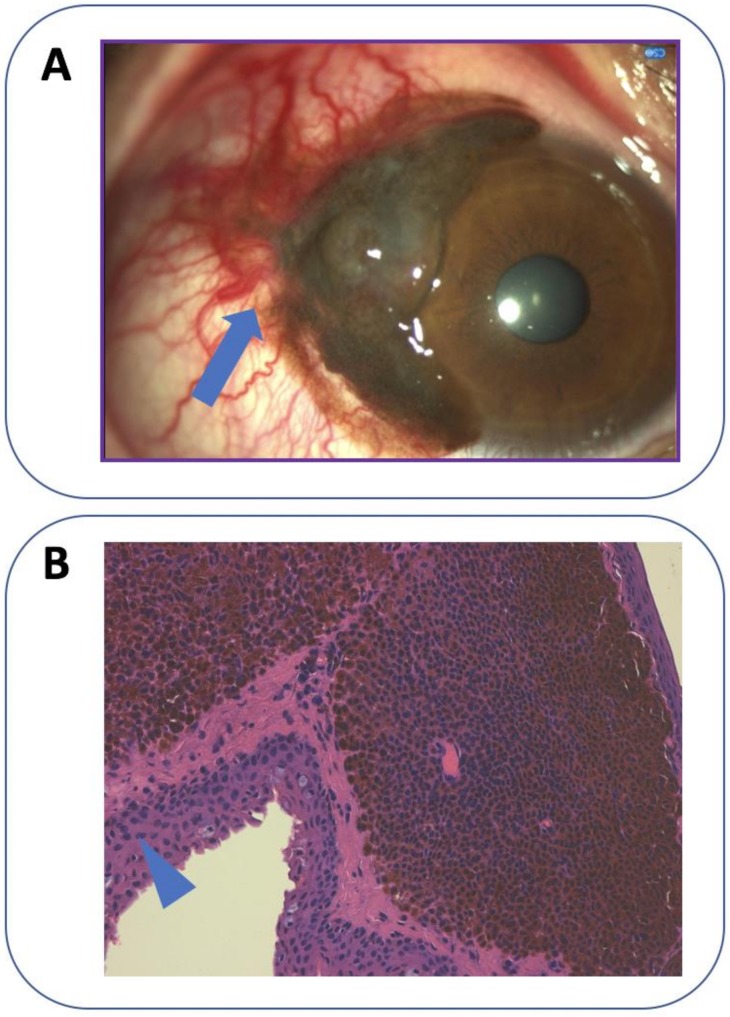
Clinical and histological appearance of melanoma of the bulbar conjunctiva. (**A**) A pigmented elevated, vascularized lesion (arrow) with feeder vessels located in the temporal bulbar conjunctiva and in the limbus. It originated in the conjunctival epithelium and gradually grew over the cornea. (**B**) Histological appearance of the lesion in (A). Dense cohesive sheets of rounded cells with varying pigmentation can be seen in the stroma, constituting a conjunctival melanoma. The overlying epithelium has normal appearance (arrowhead). Original magnification 200×—courtesy of Vincenzo Fiorentino, Pathology Department, Fondazione Policlinico Universitario Agostino Gemelli IRCCS).

Conjunctival melanoma (CjM) constitutes 5% of all ocular melanomas. It originates from melanocytes in the basal layer of the epithelium of the conjunctival membrane [1,2,3]. The incidence of CjM in Europe and the US is around 0.2–0.7 cases per million annually and this disease predominantly affects Caucasians and the elderly, while it is rare among children [3,4,5,6,7,8,9,10,11,12,13,14]. Primary acquired melanosis ‘PAM’ with atypia is responsible for up to 60% of conjunctival melanomas, with a transformation risk of about 13% [15,16,17,18,19]. ‘PAM’, also indicated as ‘conjunctival melanocytic intraepithelial neoplasia’ or ‘intraepithelial melanocytic proliferation’, presents as an acquired brown pigmentation of the conjunctiva [19]. CjM can be preceded by conjunctival nevi, which develop into melanomas in <7% of the cases [20]. CjM may also occur ‘de novo’ in about 19% of the cases [9,15,16]. It usually presents with pigmented lesions that are most commonly located on the bulbar conjunctiva (Figure 1) (92%) and, in over 60% of cases, it affects the temporal quadrants. Less frequently affected are the palpebral and forniceal conjunctiva, plica semilunaris and caruncula, which have the worst prognosis [9,15,21,22,23]. Conjunctival melanomas are multifocal in about 30% of the cases [21]. Occasionally, lesions of CjM are unpigmented [22]. CjM shows a five-year local recurrence rate from 26% to 61% [9,15,21,23,24]. Non-epibulbar lesions have the highest risk of local recurrence [14,15]. CjM spreads directly towards the orbit or through lymphatic and hematic vessels [15,25]. Temporal CjM diffuses to the pre-auricular lymph nodes, whereas the submandibular lymph nodes are usually involved in cases of nasal CjM [26]. Distant metastases are frequently found in the liver, lungs, skin and brain [9,15]. Tumors with a nodular growth pattern, recurrent lesions and ‘de novo’ CjM have the highest risk of metastatic spread [21,23]. CjM has a 10-year mortality rate of approximately 30% [3,5,6,8,15,22,27].

Genetic and epigenetic features of CjM have not been extensively elucidated to date. Some risk factors, such as sunlight exposure, and some genetic alterations are typical of both cutaneous and conjunctival melanoma. V-raf murine sarcoma viral oncogene homolog B1 (*BRAF*) V600E mutation characterizes up to 50% of conjunctival melanomas as an early event in tumor development [1,2,3,28,29,30,31,32,33,34]. *NF1* mutations can be detected in about 30% of conjunctival melanomas [35]. Neuroblastoma RAS viral [v-ras] oncogene homolog (*NRAS*) mutations occur in about 20% of the cases and are mutually exclusive with *BRAF* mutations [29,32,33,36,37]. *KIT* mutations are more seldomly detected (lower than 7%) and are mutually exclusive with *NRAS* and *BRAF* mutations [31,36,37,38]. Oncogenic signaling altered in CjM includes both MAPK and PI3K pathways. As a matter of fact, phosphorylated active forms of proteins belonging to the PI3K/AKT pathway and its downstream effector mTOR are frequently overexpressed in CjM cells, while decreased levels of phosphatase and tensin homolog (PTEN)—inhibiting PI3K/AKT/mTOR cascade—occur in this disease [39,40]. Furthermore, an increased telomerase activity with *TERT* promoter mutations can be found in about 40% of conjunctival melanomas [41,42]. In addition, molecular features of this tumor may also include the overexpression of HSP90 and Bcl-2, the inactivation of p16, other minor chromosome abnormalities and miRNAs upregulation [43,44,45,46]. However, none of these genetic or epigenetic alterations seems to have a prognostic role in CjM.

**Figure 2:**
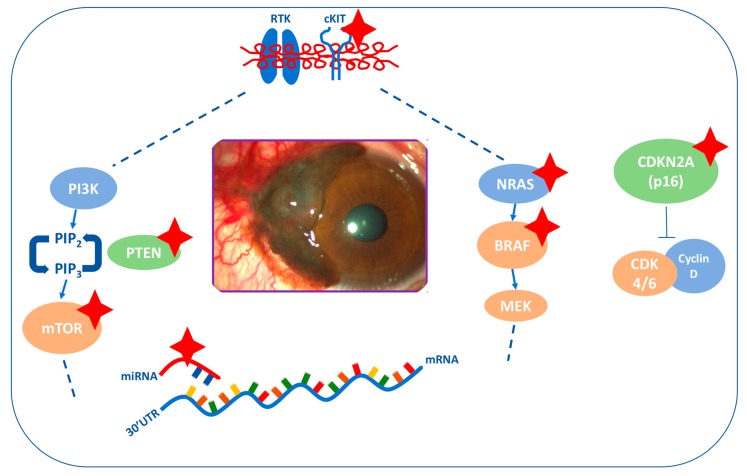
Main mechanisms involved in conjunctival melanoma (CjM). The most relevant alterations are indicated with red stars. *BRAF* and *NRAS* mutations are mutually exclusive. *cKIT* mutations are mutually exclusive with *BRAF*/*NRAS* mutations.

This review aims to elucidate in detail the genetic and epigenetic features of CjM involved in invasion and metastatic spread in order to identify potential therapeutic targets for this disease. Moreover, we aim to point out that CjM could be identified as a distinct subset of melanoma with specific genetic and epigenetic alterations that are not completely shared with other types of melanoma, such as cutaneous, mucosal or uveal melanoma (Figure 2).

## 2. Methods

The literature search was performed using electronic databases (Pubmed, Scopus and Web of Science) and selected keywords (such as “conjunctival melanoma”, “genetic”, “pathway”), linked with the Boolean operator “AND” and “OR”. Reference list of the articles was manually screened to find other relevant papers through the snowball search technique. A total of 950 full-length papers, including original researches, case reports and reviews, were identified. All the articles regarding genetic and epigenetic of CjM were considered. Papers dealing with the most important pathways involved in cutaneous, mucosal and uveal melanoma were also selected.

## 3. Genetic and Epigenetic Features of Conjunctival Melanoma

CjM has several molecular alterations associated with malignant transformation, invasion and distant spread. RAS-RAF-MEK-ERK is one of the pathways more frequently dysregulated in CjM [28]. This pathway transfers the signal from the plasmatic membrane to the nucleus, activating transcriptional factors and regulating gene expression [47]. In CjM, its activation most commonly depends on *BRAF*, *NRAS* or *KIT* mutations [48]. The frequency of *BRAF*, *NRAS* and *KIT* mutations in CjM is more similar to cutaneous melanoma than uveal/mucosal melanoma [28,29,38,48,49,50,51].

### 3.1. BRAF

*BRAF* mutations have been detected in up to 50% of primary and metastatic conjunctival melanomas as in cutaneous melanoma [1,2,3,28,29,30,31,32,33,34,51]. About 80–90% of the mutations are represented by the V600E (substitution of valine with glutamic acid, at aminoacid 600) [33,51]. The second most common mutation is V600K (substitution of valine with lysine, at aminoacid 600) [51]. Other uncommon *BRAF* mutations are detectable in <6% of conjunctival melanomas [52]. These *BRAF* mutations found in CjM are similar to cutaneous melanoma, in which V600E represents the most typical mutation (almost 70% of cases), followed by V600K (about 20% of cases) and less frequent mutations, such as V600D and V600R [52]. Acral and mucosal melanomas more rarely harbor *BRAF* mutations (respectively, 10–15% and 5% of cases) [53,54], which, on the contrary, have never been reported in uveal melanoma [55].

*BRAF* mutations are frequently associated with melanocytic nevi (up to 67%) and probably occur in early stages of CjM development from nevi [31,32,33]. Indeed, up to 50% of conjunctival nevi harbor *BRAF* mutations, which are less common in PAM [31,40].

*BRAF*-mutant conjunctival melanomas usually occur in young males and present with pigmented cells more frequently than *BRAF* wild-type conjunctival melanomas [51,56]. Similarly, in cutaneous melanoma *BRAF* mutations are more predominant among younger patients [57]. Moreover, *BRAF*-mutant conjunctival melanomas are more common on the bulbar than extrabulbar conjunctiva. This higher incidence of *BRAF* mutations in the bulbar region of the eye, more exposed to sunlight, identifies UV radiations as a possible risk factor for this disease [31,32,33].

*BRAF* mutations are not significantly associated with increased recurrence, regional metastases or mortality from CjM, but they are correlated with reduced distant metastases free-survival [33,51].

In vitro, Vemurafenib and Dabrafenib inhibit *BRAF*-mutant CjM cell lines, similarly to cutaneous melanoma cells [40]. In vivo, several *BRAF* mutated conjunctival melanomas were effectively treated with BRAF inhibitors in monotherapy or in combination with MEK inhibitors [58,59,60,61,62,63]. Thus, as patients with *BRAF*-mutant cutaneous melanoma are successfully treated with combined BRAF/MEK inhibitors, it can be hypothesized that CjM patients might also benefit from these agents. In conclusion, we believe that tests for *BRAF* mutations should be included in the management of CjM and that clinical studies with BRAF and MEK inhibitors are required in this setting [64].

### 3.2. NRAS

The *NRAS* gene encodes N-ras, a GTPase protein, which is involved in the regulation of cellular division [31]. *NRAS* mutations have been found in almost 20% of conjunctival melanomas [31,38,40,44,45]. Regarding the other types of melanoma, *NRAS* mutations have been found in about 20% of cutaneous melanomas, 5–13% of mucosal melanomas and 10% of acral melanomas. *NRAS* mutations have not been detected in uveal melanomas [65]. It is worth noting that benign cutaneous nevi can harbor *NRAS* mutations [66,67].

In CjM, *NRAS* mutations are mutually exclusive with *BRAF* mutations [31,38,40,44,45], similarly to cutaneous melanoma, in which concomitant *BRAF* and *NRAS* mutations occur in less than 0.6% of cases [68].

### 3.3. KIT

*c-KIT* mutations have been detected in almost 2–7% of conjunctival melanomas and they are mutually exclusive with *NRAS* and *BRAF* mutations, as in cutaneous melanoma [32,36,38,62]. Similarly, in cutaneous melanomas, the incidence of *KIT* mutations ranges from 5.1% for non ‘sun-exposed’ patients to 9.8% for chronically ‘sun-exposed’ patients. *KIT* mutations are more frequently detected in mucosal (about 11.5% of cases) and acral (10.8% of cases) melanomas while they have not been reported in uveal melanoma [69]. Overall, *KIT* mutations show a significant association with older age [70].

It has been reported that different ethnic groups express different rates of mutations: CjM of Chinese people expresses a higher level of *KIT* mutations (11%), but a lower rate of *BRAF* mutations (8%) than Caucasian people [50].

It is noteworthy that *KIT* mutations are not directly correlated with *KIT* gene copy number or CD117, the *KIT* gene product, expression. Furthermore, not all *KIT* regions are sensitive to pharmacological inhibition [71,72,73,74,75]. Consequently, we can assume that not all *KIT* mutations are drivers in melanomas and are not principal therapeutic targets. Partial responses to KIT inhibitors have been observed in less than 20% of patients with acral, mucosal and chronically sun-damaged melanoma patients, reporting a median overall survival of 46 weeks with imatinib and 7.5 months with dasatinib [74,75].

### 3.4. NF1

The Neurofibromin 1 (*NF1*) gene (17q11.2) encodes neurofibromin 1, which inhibits the Ras protein through the hydrolysis of Ras-bound GTP [76,77]. *NF1* mutations, leading to neurofibromin 1 dysfunction, occur in up to 30% of CjM and can be found simultaneously with *BRAF* and *RAS* mutations [35]. This co-occurrence has been also detected in cutaneous melanoma [78,79,80]. On the contrary, the co-mutation of *NF1* and *KIT* has not been demonstrated in CjM, while it has been detected in up to 32% of mucosal melanoma [81].

**Figure 3:**
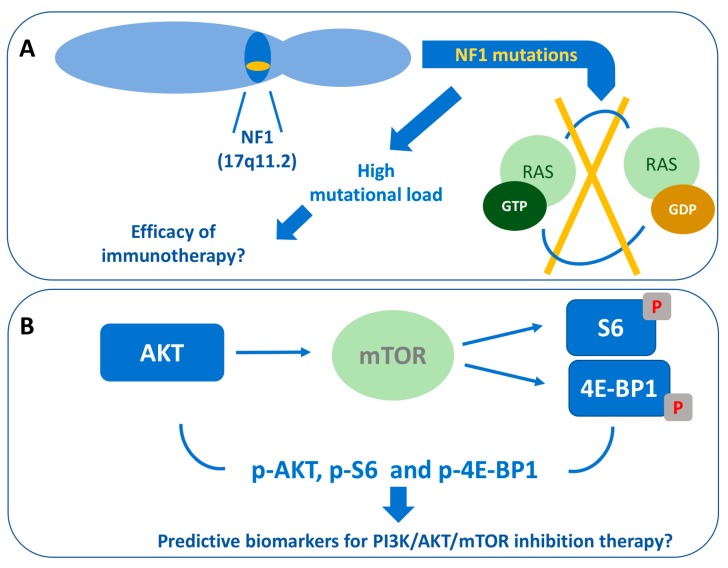
Potential molecular predictive factors for immunotherapy or target therapy in conjunctival melanoma. (**A**) *NF1* (17q11.2) mutations, which cause the loss of RAS-bound GTP hydrolysis, are associated with high mutational load and could help to predict the efficacy of immunotherapy; (**B**) High levels of AKT, phosphorylated S6 and 4E-BP1 are related to mTOR mutation and could predict the efficacy of PI3K/AKT/mTOR inhibition therapy.

*NF1* mutations are particularly frequent in CjM that have been exposed to UVs, highlighting the possible pathogenetic role of sunlight exposure [79,82,83]. *NF1* mutations are associated with sunlight exposure also in cutaneous melanoma and are more frequent in the desmoplastic subtype. It has been demonstrated that cutaneous melanomas with *NF1* mutations harbor a higher mutational load. This finding, if confirmed in CjM, could help to identify tumors which are more responsive to immunotherapy (Figure 3A) [20,84].

### 3.5. PI3K/AKT/mTOR Pathway

The PI3K/AKT/mTOR signaling pathway regulates several cellular functions, such as proliferation, metabolism, angiogenesis and metastatic spread [85]. Activated tyrosine kinase receptors (RTK), G protein-coupled receptors (GPCR) or constitutively activated Ras induce PI3K function. Class IA PI3Ks modify phosphatidylinositol-4,5-bisphosphate (PIP2) into phosphatidylinositol-3,4,5-trisphosphate (PIP3), that provides binding sites for PDK1 and mTORC2 (PDK2). These kinases activate AKT through the phosphorylation of its residues Threonine-308 (Thr308) and Serine-473 (Ser473). Factors such as PTEN antagonize AKT activation, whereas other factors, including HSP90, positively regulate AKT. After the activation, AKT phosphorylates many cytoplasmic proteins involved in cell growth and survival [85]. AKT downstream effects are mediated by mTOR, which is part of two complexes: mTORC1 and mTORC2. AKT activates mTORC1 which, in turn, inactivates the translational inhibitor 4E-BP1 and activates the kinase S6, leading to protein synthesis. mTORC2 is capable of directly activating AKT through the phosphorylation of Ser473 [85,86,87]. 

Activation of PI3K/AKT/mTOR pathway, confirmed by a high expression of phosphorylated AKT, S6, and 4E-BP1, has been reported in CjM [39]. High expression of mTOR has been found in the cytoplasm of 87% of CjM cells, and its phosphorylated form (Ser2448) in the cytoplasm and nuclei in 75% of cells. S6 and its phosphorylated form (Ser235/236) are expressed in 100% and 75% of CjM cells, respectively. The expression of 4E-BP1 and its phosphorylated form (Thr37/46) is predominantly cytoplasmic [39]. p-AKT Ser473 is mostly represented in the nuclei, while p-AKT Thr308 is both nuclear and cytoplasmic [40].

In cutaneous melanoma, the PI3K-AKT pathway is involved mostly in tumor initiation and resistance to treatments [88]. The association between mTOR nonsynonymous mutations and a short survival has been reported in cutaneous and mucosal melanoma patients [89]. In mTOR mutant cell lines, high levels of phosphorylated S6, AKT and 4E-BP1 have been found. In this context, the inhibition of PI3K/AKT/mTOR pathway exerted an antiproliferative effect [89]. Thus, the predictive value of high levels of AKT, phosphorylated S6 and phosphorylated 4E-BP1 for PI3K/AKT/mTOR inhibition therapy in CjM patients should be investigated in future studies (Figure 3B).

### 3.6. PTEN

Expression of PTEN, which is an AKT/mTOR pathway inhibitor, is low in CjM [39]. This observation strengthens the hypothesis that the mTOR pathway plays an important role in CjM development [39]. PTEN can be found in different cell compartments such as the cytoplasm and nucleus. The nuclear PTEN plays an oncosuppressive role [90,91]; it is abrogated in neoplastic cells by nuclear-cytoplasmic shuttling. Indeed, the nuclear fraction of PTEN is particularly low in CjM cells [43]. Loss of PTEN has also been observed in about 65% of cutaneous melanomas [92], while the lack of PTEN immunostaining has been reported in only 16% of uveal melanoma [93]. The hypothesis that a low expression of PTEN could allow the response to therapies with mTOR inhibitors in CjM patients should be evaluated in further studies [39].

## 4. Other Genetic Features of Conjunctival Melanoma

### 4.1. HSP90

Heat shock protein (HSP)90, a chaperone protein, plays a role in the accurate protein folding and stabilization from stress [94,95]. HSP90 expression is higher in CjM cells than in conjunctival nevi. In particular, HSP90 levels are more elevated in recurrent CjM [43]. This evidence could be particularly important for future therapies targeting specifically HSP90 [96,97]. In cutaneous melanoma HSP90 is also highly expressed, but it does not have a prognostic or predictive value [98]. In uveal melanoma, HSP90 expression has been found, but HSP90 inhibitors did not demonstrate clinical efficacy [99,100].

### 4.2. BCL-2

The B-cell lymphoma 2 (*BCL-2*) gene family encodes Bcl-2, regulatory proteins which control the mitochondrial response to apoptotic signals to preserve the mitochondrial membrane [101]. Bcl-2 can be considered a marker for melanocytic tumors in the conjunctiva [44]. It has been shown that Bcl-2 levels are remarkably higher in CjM than conjunctival nevi. Surprisingly, there was no significant correlation between Bcl-2 expression and clinical parameters or histopathological characteristics of CjM [43]. In head and neck mucosal melanoma, the high expression of Bcl-2, found in 74% of the cases, predicted a better survival [102]. Differently, in uveal melanoma the expression of protein-interacting protein 3 (BNIP3), which belongs to the Bcl-2 family, has a negative prognostic significance [103]. High levels of Bcl-2 in cutaneous melanoma are associated with aggressive behavior and metastatic spread and seem to predict chemoresistance [104].

### 4.3. TERT

**Figure 4:**
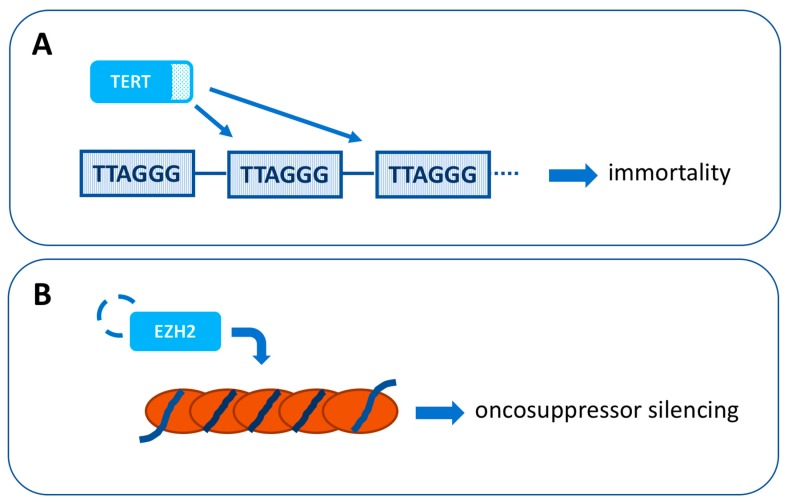
New potential therapeutic targets for conjunctival melanoma. (**A**) Telomerase reverse transcriptase (TERT) is a catalytic subunit of the telomerase enzyme that catalyzes the addition of repetitive sequences in the terminal TTAGGG of chromosomes. *TERT* promoter mutations, which cause an increased TERT expression, are detectable in 32–41% of conjunctival melanomas. (**B**) Enhancer of zeste homolog 2 (EZH2) is a histone methyltransferase which catalyses trimethylation of lysine 27 in histone H3 (H3K27me3), leading to transcriptional silencing of oncosuppressors. EZH2 is highly expressed in 50% of primary conjunctival melanomas and 88% of lymph node metastases.

Telomerase reverse transcriptase (TERT) is a catalytic subunit of the telomerase and is activated by AKT. TERT catalyzes the addition of repetitive sequences in the terminal TTAGGG of chromosomes, preventing the degradation of the chromosomal terminations with an increased cellular division rate. This mechanism leads to cellular immortality [105,106]. *TERT* promoter mutations, which cause an increased TERT expression, are detectable in 32–41% of conjunctival melanomas and in 8% of PAM cases. They have not been detected in conjunctival nevi [41,42]. We can assume that the blockage of telomeric loss consequent to *TERT* promoter mutations leads to a greater stability of the genome. Indeed, while several conjunctival melanocytic nevi harbor BRAF mutations, *TERT* promoter mutations are detectable only in melanomas and premalignant lesions (such as PAM with atypia), playing a role in tumor progression. *TERT* promoter mutations detected in CjM consist of C>T or CC>TT nucleotide changes. These alterations can be considered a typical ultraviolet (UV) effect, suggesting the potential role of UV in inducing genetic alterations involved in the pathogenesis of CjM [107,108]. The occurrence of *TERT* promoter mutations in CjM is similar to cutaneous melanoma in which *TERT* mutations can be found in 64–68% of lesions, both in primary and metastases, and are associated with a shorter survival [41,109,110]. To date, no specific prognostic role of *TERT* promoter mutations has been described in CjM. Acral and mucosal melanomas harbor *TERT* alterations respectively in up to 41% and 8% of the cases [111,112]. The detection of *TERT* promoter mutations reveals future therapeutic options for CjM. Thus, reverse transcriptase inhibitors, such as azidothymidine (AZT), may become possible candidates for therapies directed against *TERT*-promoter mutant conjunctival melanomas (Figure 4A). Imetelstat (GRN163L) is a telomerase inhibitor, which inhibits *TERT* promoter activity [113]. Other telomerase inhibitors (e.g., MST-312, TmPyP4, BIBR1532, b-rubromycin, PIPER {*N*,*N*-0-bis-[2-(1-piperidino)-ethyl]-3,4,9,10-tetracarboxylic-diimide}) have been also developed and warrant further evaluation in CjM patients [114]. 

### 4.4. CDKN2A (p16) 

Cyclin-dependent kinase-inhibitor 2A (*CDKN2A*) gene (9p21.3) encodes p16ink4a (p16) protein, which regulates the cell cycle progression through the inactivation of the complex cyclin-dependent kinase 4/6 (CDK4/6)-cyclin D [115]. p16-inactivating mutations cause the loss of its inhibitory function of CDK4/6–cyclin D complex in the G1 to the S phase transition, increasing mitotic activity [115]. *CDKN2A* mutations can be found both in cutaneous and in CjM [45,116]. Furthermore, *CDKN2A* germline mutations are associated with familiar melanomas [45,117]. Acral melanoma expresses mutations of the CDK4/6 pathway in about 82.7% of the cases [118]. *CDKN2A* alterations have been also found in mucosal melanoma of the oral cavity, but they are not related to specific clinicopathological subsets [119]. To date, *CDKN2A* mutations have never been reported in uveal melanoma [120]. 

CjM show a lower level of nuclear p16 than benign melanocytic lesions and PAMs with atypia. It has also been highlighted that lesions with thickness lower than 2 mm express higher levels of p16 [45]. In conclusion, we believe that *CDKN2A* mutations can be of interest as a potential therapeutic target for CjM and can also be useful for the differential diagnosis between CjM and benign atypical conjunctival nevi [45].

## 5. Other Genetic Alterations and Chromosome Abnormalities in Conjunctival Melanoma

Other genetic alterations have also been identified in CjM but their role in the pathogenesis of this malignancy and their significance as potential therapeutic target require further clarification.

In primary CjM, *CDKN1A* (encoding p21) and Runt-related transcription factor 2 (*RUNX2*) genes, both localized on 6p21.2, are frequently amplified [49]. p21 is a tight-binding inhibitor of CDKs and acts as a regulator of the cell cycle at the G1-S checkpoint [121]. *RUNX2* encodes a transcriptional factor that is part of the RUNT family, whose role in the metastatic process has already been investigated [122]. Furthermore, metastatic CjM conjunctival melanoma shows the amplification of *MLH1* (3p22.1) and *TIMP2* (17q25.3) and the deletion of *MGMT* (20q26.3) and *ECHS1* (10q26.3) [49]. The protein encoded by *MLH1* is involved in the DNA repair process [123,124]. *TIMP2* encodes for a matrix metalloproteinase that is critical for tissue homeostasis [125]. Deletion of *MGMT*, which is involved in genome stability, has been detected in many cancer types, including cutaneous melanoma [49]. *ECHS1* encodes an enzyme of the fatty acid beta-oxidation, but is also able to interact with STAT3 and has been found altered in many cancer types [28,49,64].

Copy number alterations (CNAs) appear more frequently in *BRAF*/*NRAS* wild-type CjM and are principally represented by losses of 1p, 3q, 6q, 8p, 9p, 10, 11q, 12q, 13, 15p and 16q, and gains of 1q, 3p, 6p, 7, 8q, 11q, 12p, 14p and 17q [31,126]. These alterations do not seem to be related to clinical features. The deletion of 10q only was correlated with shorter metastases-free survival, lymphatic invasion and major tumor thickness in 59 CjM patients [126]. Oncosuppressor genes such as *NEURL1*, *PTEN, RASSF4*, *DMBT1*, *C10orf90* and *C10orf99* are encoded from this region. Moreover, there is a higher frequency of 10q loss in *BRAF* mutant CjM [126]. In uveal melanoma, typically *BRAF*-wild type, the most frequent chromosome abnormalities, such as chromosome 3 monosomy and gain of chromosome 8q, demonstrated a prognostic value for relapse, but they did not predict response to treatment [127].

In CjM the frequency of specific chromosomal alterations varies between groups. About 30% of *BRAF*- and 43% of *NRAS*-mutant conjunctival melanomas show gains of their oncogenic loci. It is possible that a higher expression of oncogenes could play an important role in the tumorigenesis of this malignancy. Gains of 1q, 3p, and 17q occur more frequently in *NRAS*-mutant than in *BRAF*-mutant conjunctival melanomas. The loss of chromosome 10 (including *PTEN* locus) is principally detected in *BRAF*-mutant CjM. We could assume that tumors with *BRAF* mutation need a further genetic event, which induces AKT pathway, for their development. On the contrary, this additional event is not necessary for *NRAS*-mutant CjM, in which the association with chromosome 10 loss is not typical [31]. 

### 5.1. EZH2

Enhancer of zeste homolog 2 (EZH2) is a histone methyltransferase which catalyzes trimethylation of lysine 27 in histone H3 (H3K27me3), leading to transcriptional silencing of various genes, including oncosuppressors [128]. 

In the eye, EZH2 protein expression can be detected in the keratinocytes of normal conjunctiva, but it has not been found in normal conjunctival melanocytes and PAM. EZH2 is highly expressed in 50% of primary conjunctival melanomas and 88% of lymph node metastases [46]. High EZH2 is correlated with CjM thickness and poor prognosis. No correlation was found between overexpression of EZH2 and stage, local or distant relapse and tumor localization. In zebrafish xenografts, genetic and pharmacological knockdown of EZH2, through molecules such as GSK503 or UNC1999, reduces tumor growth and colony formation of CjM cells. Inactivation of EZH2 upregulates the oncogene *p21/CDKN1A*, that controls cellular transition from the G1 to S phase. Moreover, p21 levels are higher after the genetic than the pharmacological inhibition of EZH2, suggesting that EZH2 can regulate transcription using different pathways in addition to its catalytic activity [46]. Inhibition of EZH2 in CjM cells slows the cellular progression to the S-phase and determines cell death through apoptosis and autophagy. Indeed, it increases both the amount of apoptotic cleaved poli-ADP-ribose polymerases (PARPs) and the expression of LC3B-II (Microtubule-associated proteins 1A/1B light chain 3B), a hallmark of autophagy [46]. 

In conclusion, EZH2 knockdown in CjM cells leads to an S-phase depletion with G1 arrest and accumulation of cells in the G2/M phase. It derives that EZH2 prevents the death of CjM cells [46]. These findings suggest that higher EZH2 is important for tumorigenesis and progression of CjM. For this reason, EZH2 could become a therapeutic target for CjM. EZH2 overexpression has been confirmed in the other types of melanomas associated with metastatic spread and resistance to treatments [128,129,130].

The catalytic activity of EZH2 can be inhibited by some small specific target agents: The phase 1-2 clinical trial (NCT 01897571) with tazemetostat has been designed for patients with advanced solid tumors and B-cell lymphoma [46]. Further investigations are required to define the role of EZH2 in CjM development (Figure 4B).

### 5.2. miRNA 

MicroRNAs (miRNAs) are small, non-coding RNA molecules which work as epigenetic regulators causing post-transcriptional silencing of specific genes through the binding to the 30’UTR of their corresponding mRNAs [131,132]. miRNAs can play the role of oncogenes and oncosuppressors and are involved in the pathogenesis of almost all cancer types [131,133,134]. In CjM, several miRNAs are upregulated and could be considered potential prognostic biomarkers or targets for therapy [33]. Upregulated miRNAs have been also found in cutaneous melanoma [135]. MiR-20b-5p (miR-20b) has been described to be upregulated both in CjM and in cutaneous melanoma. This upregulation is responsible for PTEN suppression [33,136]. MiR-146a acts in the first phases of cancerogenesis in *BRAF*/*NRAS*-mutated cutaneous melanoma through NOTCH proteins [137]. The upregulation of miR-146a-5p (miR-146/miR-146a) and miR-146b-5p (miR-146b) has been also reported in CjM [33]. Other miRNAs upregulated both in CjM and cutaneous melanoma are miR-30d-5p (miR-30d), MiR-506-3p (miR-506), miR-509-3p (miR-509) [33,138,139]. The inhibition of miR-509 and miR-506 reduces the capability of CjM cells to grow and invade [35]. The association between the upregulation of mir-3916 and an increased risk of local recurrence of CjM has been pointed out [51]. Some miRNAs are upregulated both in CjM and mucosal melanoma [33]. Other miRNAs are also implicated in uveal melanoma metastatic spread [140].

## 6. Conclusions

Overall, CjM is commonly characterized by mutations of *BRAF*, *NF1* and *TERT*, high expression of mTOR and HSP90, frequent PTEN loss and upregulation of specific miRNAs. These alterations represent potential therapeutic targets. In particular, it could be useful to test the *BRAF* mutational status considering the high rate of mutations. The anti-BRAF and anti-MEK combination could be a therapeutic option in case of *BRAF* mutations [58].

Based on the genetic features, CjM can be considered more similar to cutaneous than mucosal melanoma and remarkably different from uveal melanoma. Consequently, we can assume that CjM is a distinct type of melanoma.

Being ocular melanomas, both CjM and uveal melanoma are often excluded from clinical trials, despite their diversity. However, taking into account the genetic profile of CjM and its similarities with cutaneous melanoma, the extension to CjM of the studies proposed for cutaneous melanoma could be encouraged.

While the knowledge of biology has improved over the last years, further information regarding genetic and epigenetic features of CjM is required to address the best targeted treatments. The challenge for the future is the identification of the driver molecular alterations to achieve a clinically relevant therapeutic effect in CjM patients.
